# This, This Is Bliss

**Published:** 1882-02

**Authors:** 


### "This, this is Bliss."

When our country was electrified, last summer, by the dastardly attack on President Garfield, when scarcely a dry eye read the last sad dispatch, or an unsympathetic ear heard the tolling of the fatal bell, how little could it be conceived that such a tragedy would be "concluded," as the play-bills have it, by such a revolting farce as Guiteau's trial has proved, or that a few months would witness a ghoul-like struggle among two or three physicians and attendants to secure a good and sufficient share of spoils from the dead. To the honor of many, and those the most eminent, who were called upon for consultation or advice, it may be said, that, their services being ended, they retired from before the public, and were no more heard of in connection with the case. But the same refinement of feeling has not, unfortunately, been displayed by all. Within a very short time one, at least, is said to have urged his claim to an amount which unblushingly announces the value he places upon his professional duties, and has calmly appropriated to himself "the lion's portion" of the expected congressional fees. To claim for himself as much as for both the distinguished consulting surgeons together, is a wand to conjure up notoriety, even more potent than formerly was condurango. No one will deny that "the laborer is worthy of his hire," nor the well-known fact that physicians, as a rule, render more gratuitous service, and experience more difficulty in obtaining their just compensation, than almost any other class of men; but the recent extravagant petition so far exceeded the legal due of the petitioner, besides giving him such unwarranted prominence above others, that it grates harshly on the feelings of a public who were led to believe, by many expressions in those days of illness, that friendship alone for the honored patient was the chief prompter of the devotion and care bestowed.

